# A comparison of *Bartonella henselae* infection in immunocompetent and immunocompromised mice

**DOI:** 10.1371/journal.pone.0297280

**Published:** 2024-02-12

**Authors:** Rebekah L. Bullard, Mercedes Cheslock, Shiva Kumar Goud Gadila, Ricardo G. Maggi, Edward B. Breitschwerdt, Ahmad A. Saied, Monica E. Embers

**Affiliations:** 1 Division of Immunology, Tulane University, Covington, Louisiana, United States of America; 2 Intracellular Pathogens Research Laboratory, Department of Clinical Sciences, Comparative Medicine Institute, College of Veterinary Medicine, North Carolina State University, Raleigh, North Carolina, United States of America; 3 Division of Comparative Pathology, Tulane National Primate Research Center, Tulane University, Covington, Louisiana, United States of America; University of Montana, UNITED STATES

## Abstract

Bartonellosis refers to disease caused by the *Bartonella* genus of bacteria. The breadth of disease manifestations associated with *Bartonella* is currently expanding and includes regional lymphadenopathy, rheumatic, ocular, and neurological disorders. The dearth of knowledge regarding diagnosis, treatment and pathogenesis of this disease can be partially attributed to the lack of a reliable small animal model for the disease. For this study, *Bartonella henselae*, the most common species associated with human disease, was injected into Swiss Webster (SW) mice. When the outcome indicated that productive infection did not occur, SCID/Beige (immune compromised) mice were inoculated. While SW mice may potentially harbor an acute infection, less than 10 days in length, the SCID/Beige model provided a sustained infection lasting up to 30-days. These data indicate that SCID/Beige mice can provide a model to study *Bartonella* infection, therapeutics, and vector dynamics in the future.

## Introduction

Bartonellosis is comprised of many disease manifestations associated with *Bartonella* spp. infections [1]. These disease states are often self-limiting to mild and require little or no medical intervention [2]. Due to the predominantly self-limiting nature of this disease, the wide array of disease states, and non-standardized methods of detection, diagnosis, and treatment, little is known about this genus of pathogens. However, the clinical definition of bartonellosis is currently expanding and includes manifestations such as angiomatoses, vasculitis, uveitis and even mental health disorders [3–6]. *Bartonella* spp. utilize vectors for transmission such as fleas, sandflies, lice, and possibly ticks [1, 7–9]. A multitude of *Bartonella* spp. have been identified in a wide variety of wild and domestic animals such as mice, shrew, rats, cats, dogs, cows, sheep, and macaques [10]. These animals may or may not exhibit clinical indicators of disease, or due to the slow-growing nature of this pathogen, infected animals may have internal masses or inflammation that go undetected clinically. The dearth of knowledge surrounding the pathogens within the *Bartonella* genus can in part be explained by the lack of a small animal model.

Comparative pathology and One Health approaches may lead to a better understanding of *Bartonella* within its multiple host populations [11, 12]. Infection with *Bartonella* species can lead to a prolonged bacteremia in the reservoir host [13–16]. Asymptomatic animal hosts may serve as a reservoir for infection of a larger population, whereby vector transmission contributes to the zoonotic infections with these bacteria [17]. Many *Bartonella* spp. have been isolated from human specimens including *B*. *koehlerae*, *B*. *clarridgeiae*, *B*. *rochalimae*, *and B*. *alsatica*. However, in the United States, *B*. *henselae* is the most common *Bartonella* species of human importance.

There has been varied success in *Bartonella* infection experiments using Swiss Webster strain (SW) mice. SW mice infected with *B*. *birtlesii* developed bacteremia lasting 9 weeks, whereas mice infected with *B*. *elizabethae* exhibited minimal infection success with only 2 of 36 mice developing bacteremia [18, 19]. One interesting note is that SW infected with *B*. *birtlesii* were inoculated intravenously and mice infected with *B*. *elizabethae* were inoculated subcutaneously. This difference could explain some of the bacteremia discrepancies [18, 19]. In another experiment, SW mice were inoculated with 3 human isolates of *B*. *tamiae* [20]. These mice were infected subcutaneously in the neck region and experienced thickening of skin at the inoculation site, which resolved in 4 weeks [20]. DNA for each isolate was amplified from tissues from the infection group and mice developed granulomas in the liver and spleen [20]. This model may be useful in evaluation of therapeutics or vector competency for *Bartonella* species. SW mice were also infected with four rat-derived *Bartonella* strains subcutaneously [21]. One induced bacteremia indicating some *Bartonella* species can switch from primary or preferred hosts to incidental hosts. These data indicate one rat-associated strain of *Bartonella* related to *B*. *coopersplainensis* may have the ability to infect mammals other than rats [21]. More investigations, however, should be performed with human pathogenic *Bartonella* species to determine whether these *Bartonella* species demonstrate different susceptibility to antimicrobial drugs compared to efficacy against rodent-derived strains. The variation in infection outcomes in the studies above may, at least in part, be due to the host immune status.

People with immunodeficiencies such as HIV/AIDS, cancer therapy, and/or immune-suppressive drugs from transplants are at a higher risk of severe manifestations [22, 23]. The immunocompetent SW mice may have the ability to eliminate the pathogen before there is damage to the host organs. Immunocompromised mice may provide a better research tool for monitoring infection and assessing bacterial spread throughout the host. In one study, researchers inoculated SCID and SCID/Beige mice with *B*. *grahamii*, *B*. *birtlesii*, *B*. *doshiae*, and *B*. *taylorii* through intraperitoneal injection [24]. These infections resulted in low-level persistence of *B*. *taylorii* in SCID mice and high-level persistence of *B*. *grahamii*, *B*. *birtlesii*, and *B*. *taylorii* in SCID/Beige mice [24]. Spleens remained enlarged at 2 months post-infection, and notable levels of pathology were recognized in the livers of SCID/Beige mice infected with *B*. *taylorii* [24]. SCID/Beige mice may serve as a model to study pathology introduced by infection and treatment response with human pathogenic *Bartonella* species, although further investigation should be performed. Studies involving immune deficient mice have implicated potential as a model, but most research surrounding immune competent mice has not explored systemic and sustained infection in *Bartonella* species that cause human disease, which will aide in the evaluation of therapeutics for human use [18, 24, 25].

For research purposes, development of a small mammal model to study bartonellosis is advantageous. Our research described here delves into the improvement of a mouse model to study infection patterns. Specific ways to determine pathogenesis in mice include: (1) observing whether *B*. *henselae* can infect mice acutely (10 days) or chronically (30 days), (2) determining whether immune status is a factor in the infection model, and (3) observing *B*. *henselae* tissue dissemination and pathology at acute timepoints (10 days) and chronic timepoints (30 days).

## Materials and methods

### Growth of *B*. *henselae*

*B*. *henselae* (San Antonio 2 strain) was grown on blood agar plates (tryptic soy agar (TSA) with 5% sheep’s blood, Fisher Scientific) at 35°C with 5% CO_2_. After 6–8 days, colonies were collected and resuspended in sterile 0.9% saline. Inoculums were made from low passage cultures (<5) to decrease the chance of changes in genetic composition.

### Mice

Female mice aged 6- to 8- weeks old (Fox Chase SCID Beige mice [strain code: 250] and Swiss Webster mice [strain code: 024]) were purchased from Charles River Laboratories and housed in group housing. Food and water were given *ad libitum* throughout the course of the experiment. SCID Beige mice were provided with sterile group housing, water, and food throughout. Experimental procedures were performed while mice were anesthetized with 3–5% isoflurane to minimize suffering. At the time of euthanasia, mice were subjected to CO_2_ until life signs were no longer present followed by cervical dislocation. Practices in the housing and care of mice conformed to the regulations and standards of the Public Health Service Policy on Humane Care and Use of Laboratory Animals, and the Guide for the Care and Use of Laboratory Animals. The Tulane National Primate Research Center (TNPRC) is fully accredited by the Association for the Assessment and Accreditation of Laboratory Animal Care-International. Animal work was approved by and conducted under the supervision of Tulane’s Institutional Animal Care and Use Committee.

### Injection and monitoring of infection

Mice were anesthetized with isoflurane and injected subcutaneously with 200uL of inoculum (10^6^ cells/mL in sterile 0.9% saline). Sets of mice (n = 2 controls, 3 experimental each) were humanely euthanized and necropsied at days 10, 20, and 30. Mice were perfused with ice cold 4% paraformaldehyde before tissue removal. Up to 15 tissues were taken at the time of necropsy: brain, ears, thyroid, thymus, heart, lung, liver, kidney, adrenal glands, pancreas, spleen, bladder, tibiotarsal joint, lymph nodes, and bone marrow. Four tissues (brain, heart, liver, kidney) were divided for DNA and pathology. Samples for pathology were placed in cassettes and stored in zinc formalin fixative (Z-fix). Culturing organs in Grace’s insect media from unfixed tissues was attempted but was unsuccessful.

### Genomic DNA (gDNA) extraction and PCR

Tissue samples were frozen at -20°C until ready to use. Samples were thawed on ice and a small sample was taken from each and placed in a separate tube. Qiagen DNeasy Blood and Tissue Kits (Qiagen) were used to isolate gDNA. DNA concentration was measured on a Nanodrop Spectrophotometer and stored at -20°C. PCR was performed on all samples using nested vOmp1 primers. (External primers: Bh197676ext FWD—5’ ATTATGCAATTGCTATAGGTTTAGAAGCTGA 3’; Bh198246ext RVS—5’ CAAAGTGAATTCAATTTTATGCTTATTATCTACAG 3’; Internal primers Bh197940int FWD—5’ ACGATCCTGCAGCAAATGCTCCGATAA 3’; Bh198112int RVS—5’ CAGAAATTTTCCAAGCCCCTGCTACCACATCT 3’). Reactions were prepared using Taq polymerase (Qiagen) with the following concentration: 2.5 units *Taq* DNA polymerase, 1.5mM MgCl_2_, 200μM of each dNTP, 0.4μM each primer, 250ng gDNA template. Internal reactions contain the same concentration of reagents but take 5μL of the external reaction as the template. External reaction had thermocycling conditions of 95°C for 3 min; 35 cycles of 95°C for 30s, 63°C for 30s, and 72°C for 30s; followed by 72°C for 5 min. The internal reaction utilizes the following thermocycling conditions are used: 95°C for 3 min; 35 cycles of 95°C for 30 and 72°C for 30s; followed by 72°C for 5 min. PCR products were visualized on 2% agarose gel with 0.1% ethidium bromide.

### Histopathology

Fixed tissues were cut into 5μm sections and mounted onto slides. Slides were stained with hematoxylin and eosin (H&E) stain and examined by a board-certified veterinary pathologist. Samples were scored on a scale of 0–3 based on the severity of pathology noted: 0 = no significant findings, 1 = minimal findings, 2 = mild findings, and 3 = moderate findings.

## Results and discussion

### gDNA from *B*. *henselae* can be identified in multiple tissues of SCID/beige mice but rarely in SW mice

Mice were examined for *B*. *henselae* gDNA using nested PCR of the vOMP1 gene. On day 10, one of 27SW mouse tissues, the heart, was PCR positive. Based on these data, it appears that *B*. *henselae* does not develop a productive infection with SW mice. Most mice (all mice in some groups) were *Bartonella* PCR negative (Table 1). *Bartonella* gDNA was not identified in any of the control tissues from any of the uninfected mice.

**Table 1:** Tissue distribution of *Bartonella henselae* gDNA in Swiss Webster and SCID/beige mice.

	Tissues										
	Timepoint (n = 3)	Ear	Heart	Bladder	Spleen	TTJ	LN	Brain	Liver	Bone Marrow	Total
Swiss Webster	Day 10	0	1	0	0	0	0	0	0	0	1^a^
	Day 20	0	0	0	0	0	0	0	0	0	0^b^
	Day 30	0	0	0	0	0	0	0	0	0	0^b^
Total SW		0	1	0	0	0	0	0	0	0	1
SCID/beige	Day 10	0	1	0	1	0	0	0	0	0	2
	Day 20	0	0	1	0	0	1	1	0	0	3
	Day 30	1	0	2	0	1	0	3	0	1	8^a^^,^^b^
Total SCID/beige		1	1	3	1	1	1	4	0	1	13

^a^ Statistical significance p = 0.0380

^b^ Statistical significance p = 0.0120

Two-way ANOVA Tukey’s multiple comparisons test

When determining whether immune status is a factor in the infection model, it can clearly be seen that the immune compromised model was successful, whereas the immune competent model was not. A total of 13 tissues were positive for gDNA across all the time points in the SCID/beige group. More tissues containing *B*. *henselae* gDNA were identified as time from initial infection progressed, with at least one tissue in each mouse containing gDNA by day 30. The eight tissues containing *B*. *henselae* gDNA in the SCID/beige mice euthanized at 30 days post-infection was statistically more than the total number of SW tissues from any of the previous time points (Table 1). *Bartonella* DNA was amplified from at least one tissue in all except for two SCID/beige mice upon necropsy. It is interesting to note that 4/9 (all data points) and 3/3 (D30) brains were positive for *B*. *henselae* DNA. However, *B*. *henselae* DNA was not identified in the livers of the mice injected with *B*. *henselae* and only in the heart of one mouse and the kidneys of two mice. Seven out of nine mice were infected using the SCID/Beige mice, resulting in significantly more mice infected than the SW model.

**Fig 1:**
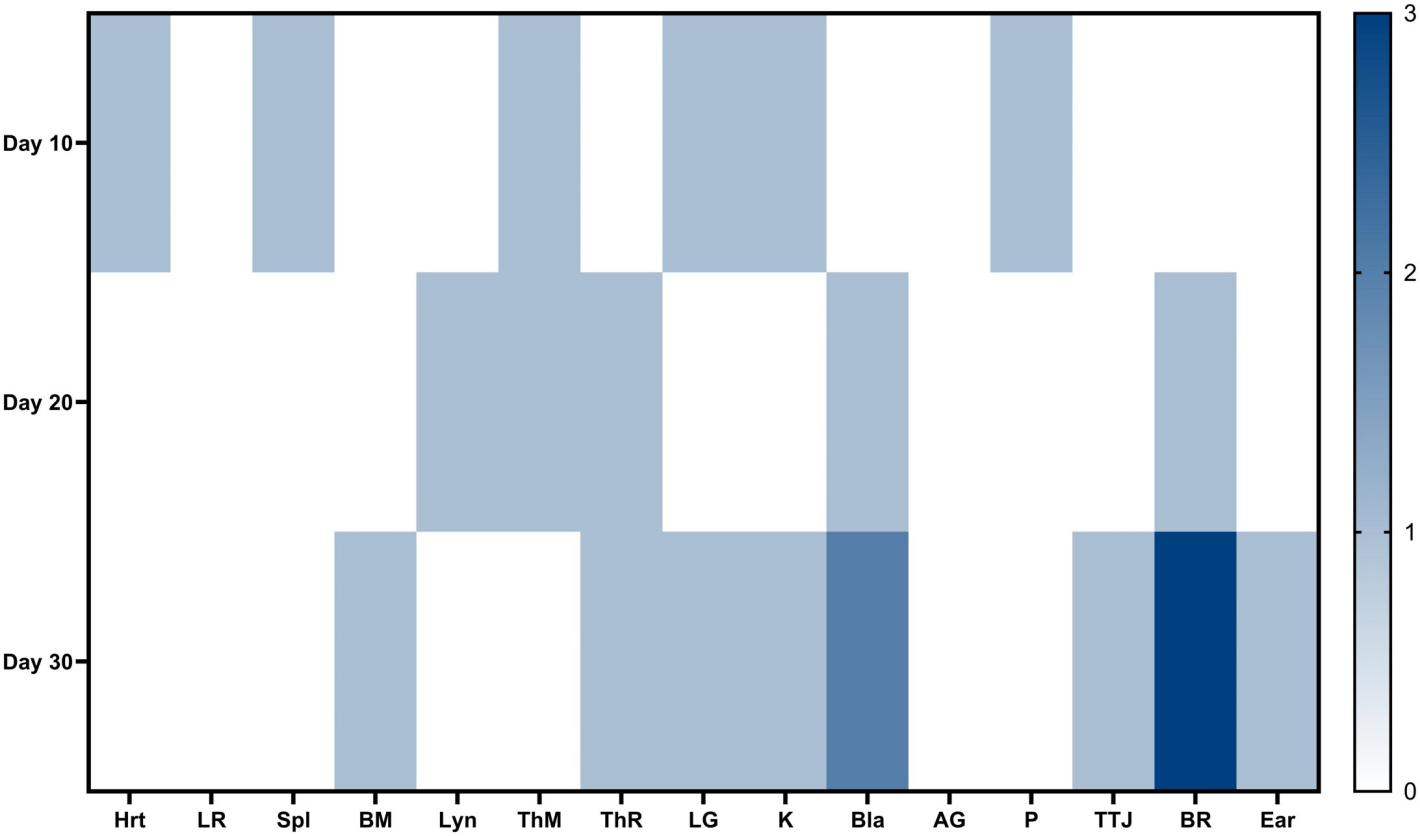
Heatmap of the tissue distribution of PCR positive tissues in SCID mice at 10-, 20-, or 30-days post-infection. The bladders and brains of 2/3 mice and 3/3 mice were positive for infection, respectively, at 30-days post-inoculation. Other patterns remain unclear as only one mouse each was positive for the other tissues. n = 3 for each time point. Hrt = heart, LR = liver, Spl = spleen, BM = bone marrow, LyN = lymph node, ThM = thymus, ThR = thyroid, LG = lung, K = kidney, Bla = bladder, AG = adrenal glands, P = pancreas, TTJ = tiobiotarsal joint, BR = brain, Ear.

Immune compromised mice have been used in the past as a *Bartonella* model, so these data are not strikingly different than what has been previously published [24]. However, we could assess whether infection resulted in acute or chronic infection. In this case, we observed tissue distribution for bacterial dissemination. Although tissue distribution did increase from 10-days post-infection to 20-days-post infection, bacteria did not appear to further disseminate (Table 1; Fig 1). While none of these differences were statistically significant, bacteria were disseminating over time. These data indicate that SCID/Beige mice develop an acute and chronic infection with *B*. *henselae*.

This may also indicate that infection may be occurring in the SW mice, but the resulting infection is extremely acute and resolved before the 10-day timepoint. The acute window for these *Bartonella* species could be as short as 2–5 days of infection for SW mice with the infection terminated by the immune system. It is also possible that these *Bartonella* species were not given enough time to cause a patent infection in SW mice. Some experiments found that it took multiple weeks for bacteremia to be established with *Bartonella* in mice. It is possible the bacteria need a longer incubation period within the primary niche before dissemination occurs.

Prior studies have also indicated that enrichment with Bartonella Alpha-proteobacteria growth medium (BAPGM) may be necessary to grow the bacteria to a level that is detectable by PCR. In the study presented here, we attempted to culture the bacteria from tissues using Grace’s media due to supply issues regarding BAPGM. Although we were ultimately unable to culture viable bacteria from the tissues, the presence of living bacteria cannot be ruled out. The presence of red blood cells in this media enhances *Bartonella* growth from tissues when compared to other liquid media types known to support *Bartonella* growth such as Grace’s media. BAPGM has been used to isolate different *Bartonella* species from humans, dogs and cats [26]. However, it has yet to be applied to mouse models [26–29].

Although suspected, the primary niche of *Bartonella* is not known [30]. It is possible it was not obtained in these mice and could include a tissue such as the lung, where *Bartonella* has been found before [31–33]. As stated above, based on these data SW may result in an acute infection that is terminated by the immune system. Another important aspect to consider is that since *Bartonella* species are transmitted by vectors, a component to allow bacterial persistence is missing. Vector saliva has a multitude of components that allows for saliva-assisted transmission (SAT) [34–36]. Characterized components include vasodilators, immune modulators, and antihistamines that may be imperative for *Bartonella* to establish an infection [34–37]. Other studies have shown that mice can be infected with *B*. *birtlesii*, using *Ixodes ricinus* [38]. It may be that vector competency is instrumental when attempting to infect immune competent mice. The lack of natural infection may also interfere with the protein expression and overall structure of the *B*. *henselae*. A previous study examined changes in the structure of *B*. *henselae* (Houston 1) from *in vitro* cultured bacteria and the same strain harvested from infected mice. This study identified changes in relative abundance of the bacteria as well as changes to the outer surface with a reduction of fimbriae in colonies grown from mouse blood. These structural changes may play a significant role in the histopathological findings of both the Velho et al. study [39] and the present data.

### Immunocompetent mice exhibit pathological changes in the liver and kidneys

To determine if the mice developed any degree of disease or altered function due to the infection, the hearts and brains of SW and SCID/beige mice infected for 30 days were fixed in Z-fix and processed for histology. None of the brains or hearts had significant pathological findings despite all the SCID/beige brains containing *B*. *henselae* gDNA (Tables 2 and 3).

**Table 2:** Pathology and PCR results from *B*. *henselae*-infected SW mice.

Mouse Identity	Pathology Score	
	Heart	Brain
WE55	0	0
WE56	0	0
WE57	0	0
WE58	0	0
WE59	0	0

**Table 3:** Pathology of SCID/Beige mice infected with *B*. *henselae*.

Mouse Identity	Pathology			
	Heart	Brain	Liver	Kidney
WG31	0	0	0	0
WG32	0	0	2	0
WG33	0	0	1	0

**Fig 2:**
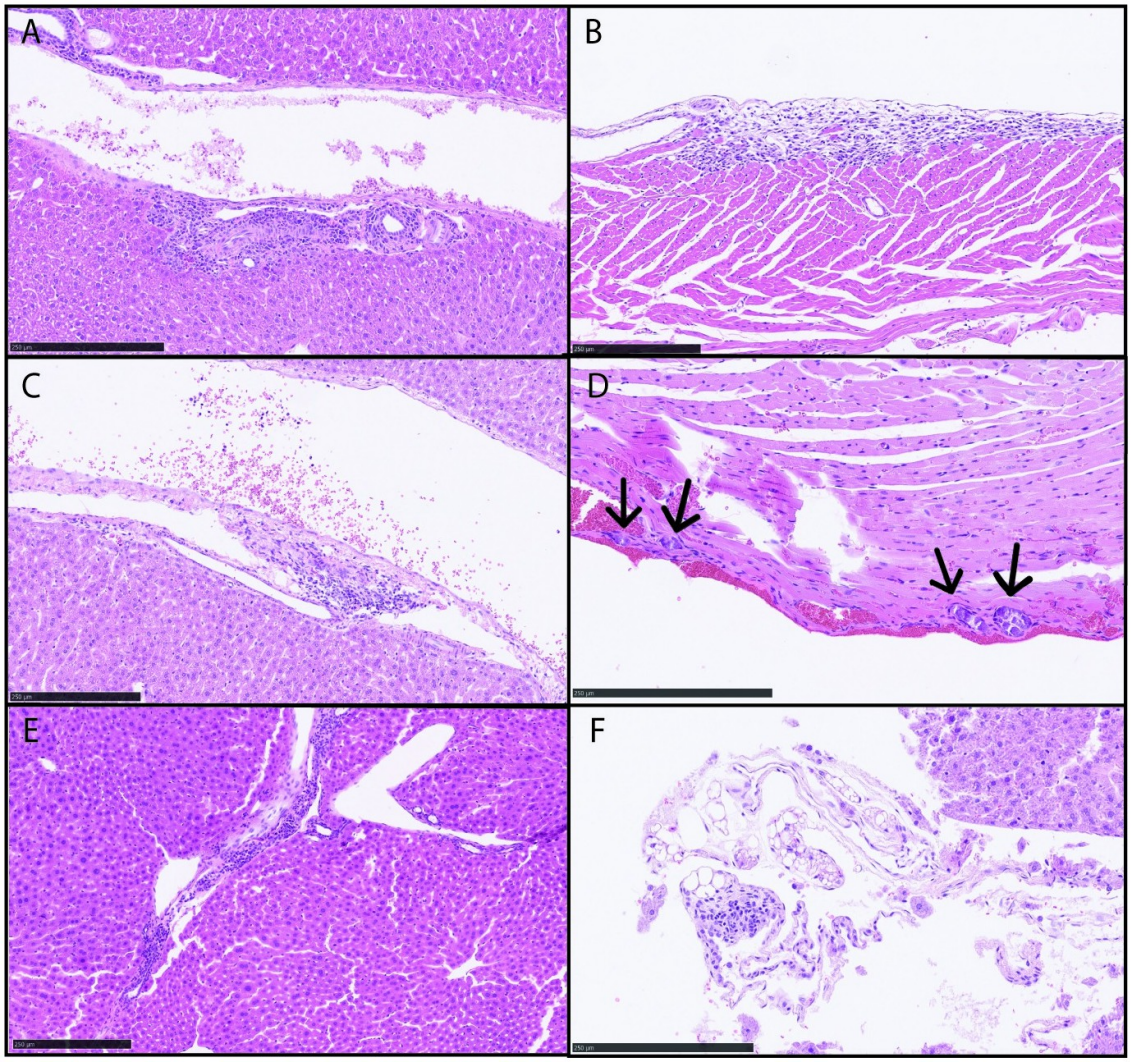
Histopathology of tissues from SCID/beige mice infected with *B*. *henselae*. (A, B) Mice were necropsied 10 dpi and tissues were stained with H&E stain for histopathological analysis. (A) Liver sections were found to contain mild multifocal portal mononuclear and suppurative infiltrations; (B) Heart sections contained focally extensive, moderate mononuclear epicarditis with extensions into the myocardium. (C,D) Tissues from 20 dpi with *B*. *henselae*. (C) Liver sections contained mild multifocal portal mononuclear infiltrations with minimum extramedullary hematopoiesis; (D) Heart sections contained minimal multifocal mineralization of the blood vessels (arrows). (E,F) Tissues from mice 30 dpi with *B*. *henselae*. Livers from two different mice contained (E) mild multifocal portal mononuclear inflammation and (F) focal granuloma in the peritoneal fat with minimal portal mononuclear infiltration. All scale bars = 250μm.

Immunocompromised patients who have become infected with *Bartonella* spp. often show symptoms related to kidney and liver pathology in the form of bacillary angiomatosis and peliosis hepatis. For this reason, we harvested these tissues from immunocompromised mice for histological analysis. After 30 days of infection, two of the three mice had pathological lesions (Fig 2). Additionally, the immune compromised nature of this strain of mouse made them more susceptible to pathological changes earlier in the infection process. Granulomatous hepatitis has been reported in humans, a dog, and a horse infected with *B*. *henselae*. Hepatic manifestations of *B*. *henselae* often include irregular microabscesses surrounded by histiocytes, lymphocytes and a layer of fibrous tissue. Other manifestations include small granulomas with giant cells and small foci of necrosis [40]. The immune compromised model described here further substantiates this pathology [41, 42]. Heart, brain, liver, and kidneys were collected from mice infected for 10- and 20-days and prepared for histological analysis. Table 4 compares the presence of *Bartonella* gDNA to changes in pathology from all SCID/beige mice. When compared in this manner, only one mouse (WG33) had histological differences in the same tissue that contained *Bartonella* gDNA; however, only a minimal mineralization of a blood vessel was noted that is likely an artifact from the necropsy or staining process (S1 Fig) *Bartonella* homing to the brain may be an important component in pathogenesis and may help us understand neurological symptoms occurring in human patients in the future. Although brain was positive for *B*. *henselae* DNA by PCR, there were no lesions or irregularities within the brain sections used for pathological analysis. It is possible that a low bacterial burden or length of infection was not sufficient to produce pathology in this location.

**Table 4:** Comparison of PCR and histological results of SCID/beige mice.

Day post infection	Mouse number	PCR + in any tissue	Tissue	PCR+	Histopathology Lesions
Negative Controls	WG22-24 WG58-59		Brain		
			Heart		
			Liver		
			Kidney		
10	WG25	x	Brain		
			Heart	x	
			Liver		x
			Kidney	x	
	WG26	x	Brain		
			Heart		
			Liver		
			Kidney		
	WG27		Brain		
			Heart		x
			Liver		
			Kidney		
20	WG28	x	Brain	x	
			Heart		
			Liver		
			Kidney		
	WG29		Brain		
			Heart		
			Liver		x
			Kidney		
	WG30	x	Brain		x
			Heart		
			Liver		
			Kidney		
30	WG31	x	Brain	x	
			Heart		
			Liver		
			Kidney		
	WG32	x	Brain	x	
			Heart		
			Liver		x
			Kidney		
	WG33	x	Brain	x	
			Heart		
			Liver		x
			Kidney	x	

At time points prior to 30-days post infection, histopathological changes can be identified in the hearts of two mice. One mouse was found to have mononuclear epicarditis and the other had multifocal mineralization of the blood vessels that were not considered to be artifacts of the staining process. In human cases, *B*. *quintana* is more closely related to infectious endocarditis. However, one case study found vegetation of the aortic valve and an abscess near the mitral valve interfering with its function. Histopathological examination revealed infiltrates of mononuclear cells and macrophages, vascular neoformation, and fibrosis [43]. These findings are more advanced but still correlate to the findings in the mouse heart tissues.

## Conclusions

Overall, our data indicate a limited model to study bartonellosis within a mouse. The use of SCID/Beige mice may help explore questions about infection and treatment in relationship to *B*. *henselae*. While antibiotic susceptibility has been studied *in vitro*, the use of a mouse to study *in vivo* results can validate and assist with discerning treatment regimen efficacy for people experiencing serious disease manifestations [44].

An important consideration with these experiments is the use of a needle to inoculate the mice. In nature, the source of infection is likely a hematophagous vector, such as a flea, tick, or other vector [45, 46]. However, needle stick transmission of *Bartonella* to veterinarians from an infected cat or dog supports transmission of virulent bacteria directly from an infected host. For future studies, the use of a vector in facilitating infection is recommended and may improve our understanding of the significant role a vector plays in transmission to mammals. With such ubiquity within animals and vectors, and the extensive manifestations surrounding bartonellosis, developing an animal model will open opportunities to study the many mysterious qualities of disease, such as vector competency, improved diagnostic modalities, and effective therapeutics for patients infected with *B*. *henselae*.

## Supporting information

S1 Fig Kidney from a mouse 30 dpi *Bartonella henselae* infection. Arrow indicates the formation of visible mineralization. This type of mineralization is considered to be an artifact of the staining process and not a true pathology.
(TIF)
