# Supplementary figures and images for: A comparison of Bartonella henselae infection in immunocompetent and immunocompromised mice
Source: PLoS One. 2024 Feb 12;19(2):e0297280. doi: 10.1371/journal.pone.0297280 (PMC10861063; doi:10.1371/journal.pone.0297280)

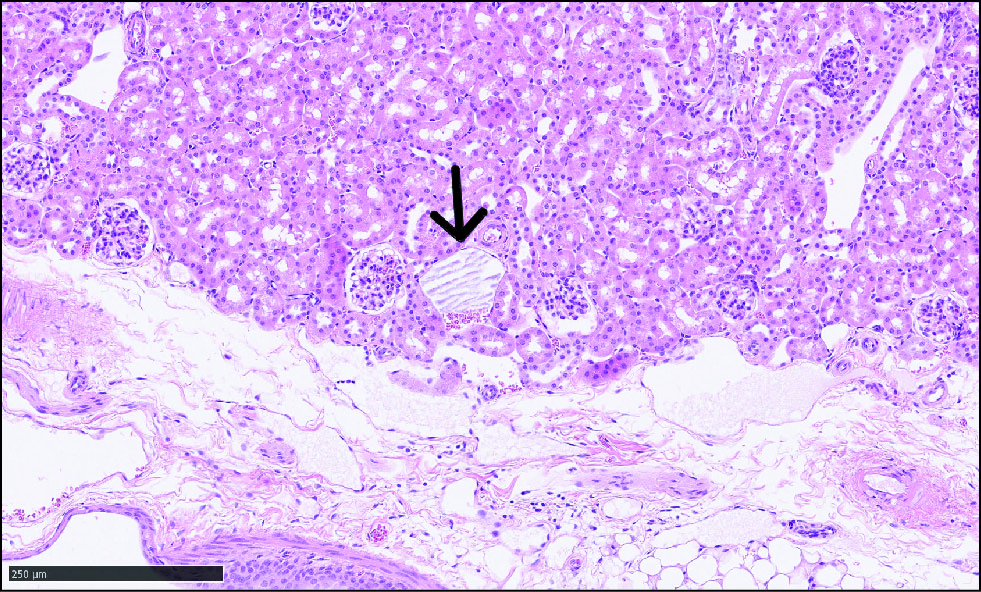

Supplement: S1 Fig — Arrow indicates the formation of visible mineralization. This type of mineralization is considered to be an artifact of the staining process and not a true pathology. (TIF) [file pone.0297280.s001.tif]
